# Randomized Double-blind Placebo-controlled study to evaluate the efficacy of fermented deglycyrrhizinated licorice for treatment of diabetic polyneuropathy

**DOI:** 10.1007/s12020-025-04476-5

**Published:** 2026-02-02

**Authors:** Ahmad Mohamed Ali Massoud, Hoda Mohamed Ali Massoud, Ahmed Abdel-Aala El-Shawarby, Mahmoud Ibrahim Elseidy

**Affiliations:** 1 Department of Hepato-gastroenterology and Infectious Diseases, Faculty of Medicine, Al-Azhar University, Cairo, Egypt; 2 Department of Neurology, Faculty of Medicine for girls, Al-Azhar University, Cairo, Egypt; 3 Department of Anesthesiology, Faculty of Medicine, Ain Shams University, Cairo, Egypt; 4 Department of Pharmacy Practice, Faculty of Pharmacy, Helwan University, Cairo, Egypt

**Keywords:** Diabetic polyneuropathy, FDGL, Licorice, Diabetes complications, Type 1 diabetes, Type 2 diabetes

## Abstract

**Purpose:**

To evaluate the efficacy of α-amylase enzyme prepared from fermented deglycyrrhizinated licorice root extract (FDGL) in treating early-stage diabetic polyneuropathy (DPN).

**Methods:**

A double-blind, placebo-controlled randomized trial involving 83 type 1 and II diabetic patients aged 15–65 years with diabetes duration ≤ 5 years, serum amylase < 45 IU/L. Patients were not complaining of clinical diabetic polyneuropathy (DPN), yet they showed demonstrable electrophysiological changes (vibration perception threshold [VPT] > 15 V), low nerve conduction velocity (NCV), and low serum amylase levels. Patients were randomized to receive either FDGL capsules (gp A; 0.5 gm twice daily containing 1250 IU of α-amylase) or a matched placebo (gp B) for 6 months. For comparison, 20 healthy volunteers matched for age, sex, and body constitution were recruited (control gp). Primary outcomes were the changes in motor nerve conduction velocity (MCV) and vibration perception threshold (VPT) from baseline. Secondary outcomes included changes in sensory nerve conduction velocity (SCV), serum amylase levels, and HbA1C. Outcomes were assessed at baseline, after 3, and 6 months. The study was registered on ClinicalTrials.gov (NCT07148804).

**Results:**

A total of 83 diabetic patients were analyzed. Compared to placebo, patients who received FDGL (GpA) demonstrated a significant increase in MCV, SCV, and serum amylase with a reduction in VPT at 3 and 6-month follow-up (*p* < 0.001 for all outcomes). Compared to the control group, this corresponds to a restoration of 32% and 99% of the neurological deficits of MCV and SCV, respectively. HBA1C remained around the baseline values at the end of the study with no significant difference between the groups.

**Conclusion:**

Oral administration of α-amylase enzyme in FDGL significantly improved nerve conduction and restored serum amylase levels in both type I and II diabetic patients. Further studies are needed to verify its effect on other complications.

**Clinical trial registration:**

This study was registered at ClinicalTrials.gov (NCT07148804).

**Supplementary Information:**

The online version contains supplementary material available at 10.1007/s12020-025-04476-5.

## Introduction

Studies concerning insulin resistance syndrome, which is closely linked with type II diabetes mellitus (DM), suggest that the underlying cause is a metabolic error in the mitochondria, which limits energy liberation from the breakdown of glucose [1, 2]. If we take into consideration that the degradation of hexoses both in the cytoplasm (glycolysis) and in the mitochondria (tricarboxylic acid cycle, TCA cycle and the respiratory chain) depends entirely on a cascade of enzymes, and these enzymes represent the link between the activated insulin receptor (as a start point) and the mitochondrial energy liberation (as an end point), it would be reasonable to presume that this error is an enzymatic defect somewhere in this cascade of enzymes. Amylase enzyme is a glycolytic enzyme produced by the exocrine pancreas to help digest starch in the gut lumen. It is then absorbed from the gut in an intact form by a macromolecular protein absorption mechanism [3], apparently to be recycled to the pancreas in what is known as the entero-pancreatic cycle of the enzyme [4, 5]. Serum amylase deficiency (which has been shown by several studies [6–8] to correlate well with the severity of diabetic complications) is thought to be the cause of the enzymatic error mentioned above. Hypoamylasemia has also been reported in type 1 DM [6, 8]. A study tracking abnormal glycemic control in infancy (4–7 months) in susceptible high-risk children for type 1 DM showed that the pancreatic insult preceded the appearance of serum autoantibodies, and that metabolic shifts were present much earlier than was expected in these children [9]. The fact that these shifts (as they reported) were coupled with a small-sized pancreas and decreased levels of glucokinase enzyme (secondary to low amylase) strongly suggests that the original insult involved both endocrine and exocrine functions of the pancreas [10]. Additionally, the fact that clinical type 1 diabetes may take years to appear may suggest that hypoamylasemia may play the same role as in type 2 diabetes. With confirmed hypoamylasemia and hypoinsulinemia, the pathogenesis of diabetic complications is logically the same in both types.

Accordingly, FDGL, a drug formulation rich in α-amylase enzyme, was prepared to replace the deficient enzyme in diabetic patients, to restore normal carbohydrate metabolic pathways, and thereby treat associated complications. Since DPN is by far the most common diabetic complication, it was selected as an appropriate model to evaluate the therapeutic impact of this formulation. Several studies have demonstrated that the underlying pathology of DPN is primarily driven by uncontrolled intracellular hyperglycemia [11, 12]. Persistent hyperglycemia stimulates the aldose reductase enzyme, leading to excessive production of sorbitol, which imposes an osmotic burden on neurons and results in neuronal dysfunction [11]. Hyperglycemia also enhances the synthesis of protein kinase C, which induces chronic neural ischemia through thickening of the arterial basement membrane and increased vascular permeability [12]. Structural neuronal damage further arises from the formation of advanced glycation end products (AGEs) through non-enzymatic reactions between excess intracellular glucose and cellular proteins, nucleotides, and lipids. This process disrupts neuronal integrity and impairs repair mechanisms [13]. In addition, hyperglycemia activates the polyol pathway, generating cytotoxic reactive oxygen species (ROS) intermediates, including superoxides, which contribute to cellular injury and necrosis [11]. From the above, it can be deduced that insulin therapy alone may aggravate the intracellular damage by continuously driving glucose into the cells, thereby worsening intracellular hyperglycemia despite lowering serum glucose levels. Therefore, a proposed strategy to counteract this mechanism is to promote glycolysis, thereby reducing intracellular glucose concentration. The amylase-enriched formulation FDGL was developed to test this hypothesis. After conducting an animal study to ensure the validity and safety of this formulation, this study was carried out [14–16]. The significant improvement of histological parameters in animal models with retinopathy [15] and nephropathy [16] suggests that these microvascular complications share common pathogenic mechanisms with neuropathy, which appear to be mitigated by FDGL treatment.

Therefore, this study aimed to assess the role of the oral drug formulation under trial comprising α-amylase enzyme (2500 IU/gm powder) in addition to flavonoids (naturally occurring in the crude licorice root) in the treatment of diabetic complications.

## Research design and methods

### Study design

This study was a double-blind, placebo-controlled study conducted at Al-Azhar University Hospitals (Nasr City, Cairo, Egypt). Patients were randomized to receive either FDGL as 500 mg capsules twice daily (Gp A; 43 patients) or placebo capsules of non-fermented deglycyrrhizinated 500 mg licorice capsules twice daily (group B; 40 patients). The control group (Gp C) of 20 healthy volunteers underwent baseline neurophysiological and biochemical assessments, but they did not receive study medication, nor did they undergo further follow-up. Their values served as reference benchmarks for normalization analyses. Placebo capsules were specifically prepared to match the color and the appearance of the active FDGL capsules. Since FDGL is an investigational medication, capsules were prepared specifically for research purposes in the laboratories of the national research center (NRC, Cairo, Egypt). The formulation was developed under controlled laboratory conditions to ensure batch uniformity and standardization. Each 500 mg capsule contained fermented deglycyrrhizinated licorice root extract providing approximately 1250 IU of α-amylase together with naturally occurring flavonoids. The preparation was used exclusively within this clinical study.

The study was approved and consistent with the institutional Human Ethics Committee and the Egyptian Committee of Medical Ethics (Approval no. 6016 A) following the precepts of Helsinki declaration and the guidelines of good clinical practice.

### Participants

Out of 269 patients with type 1 and 2 DM attending Al-Azhar University diabetic Clinic, 120 patients were chosen. Inclusion criteria comprised: age between 15 and 65 years, diabetes duration 1–5 years, BMI < 30 kg/m², HB A_1_C < 9%, serum creatinine < 2 mg/dL and serum amylase < 45 1U/L (ref. range 28–100 1U/L) and clinically asymptomatic with electrophysiological signs of early-stage DPN, as evidenced by vibration perception threshold (VPT) > 15 V. VPT has been reported to be a reliable and sensitive test for detection of DPN [17, 18].

The patients were not complaining of any symptoms of DPN and were not on treatment that might influence nerve function, e.g. antiepileptic agents, tricyclic antidepressants, nor were they on treatment with sympathomimetic agents. Patients with myopathies, neuropathies, or malignancies were also excluded. Furthermore, patients suspected of having other causes of neuropathy were excluded, including but not limited to vitamin B12 deficiency, hypothyroidism, chronic alcohol consumption, autoimmune or connective-tissue diseases, HIV infection, renal failure, or current use of neurotoxic drugs (including isoniazid, chemotherapy). Patients were explicitly instructed not to take any medication in the future before counselling the trial investigators. A total of 102 patients eventually consented to participate in the study after a detailed and comprehensive explanation of the nature of the procedure.

Twenty healthy volunteers matched for age, sex, and body constitution served as controls. The control subjects underwent the same (sensory function and neurophysiological examination, and tests as the patients at baseline.

### Randomizations

Eligible patients were randomized in a 1:1 ratio between the intervention and placebo groups using computer-generated random number generation with a block size of 4, stratified by baseline HbA1C level (< 7.5% vs. ≥ 7.5%). An independent statistician generated the randomization sequence, which was concealed using sealed, sequentially numbered, and opaque envelopes. Randomization was performed using “blockrand” package of R software (version 4.3.1, R Foundation for Statistical Computing). Both participants and investigators were blinded for treatment assignment, and the placebo capsules matched FDGL capsules for appearance, weight, and excipient composition.

### Study procedures

After signing informed consents, patients underwent a physical examination including a neurological evaluation, blood pressure measurement, and clinical lab testing, including a 2-hour postprandial serum amylase measurement. Although early DPN does not involve autonomic neuropathy, the electrocardiogram (ECG) was also performed as a part of the routine investigations.

Neurological examination was performed to confirm the absence of clinical signs of DPN and included sensory testing for pain, touch, and temperature on the big toes and on the dorsum of the feet and tibial regions. The examination also included ankle and knee reflex testing and joint proprioception for the big toes.

Furthermore, a neurophysiological examination was carried out, including nerve conduction velocity (NCV) and VPT measurement, as will be explained in detail. The patients found to fit the inclusion criteria were initially equally randomized to either of the treatment groups. The patients were instructed to take their capsules one hour before meals, 2 times daily, along with their regular antidiabetic medications. Serum glucose level was checked 2 h. later, twice daily for the first three days, and antidiabetic medication doses were adjusted in order to maintain optimal glycemic control (around the pre-study level). Once achieved, the adjusted dose was maintained thereafter. After 3 months, reassessment of all tests was performed to ensure the absence of complications, and after 6 months, the final readouts were recorded, and the study was ended. Serum amylase level was checked once for the control group and every 4 weeks in the study groups by an independent analyst during the study period and was taken as a test of patient compliance as well as for the efficiency of the drug in raising serum amylase level.

### Neurophysiological assessment

**Fig. 1:**
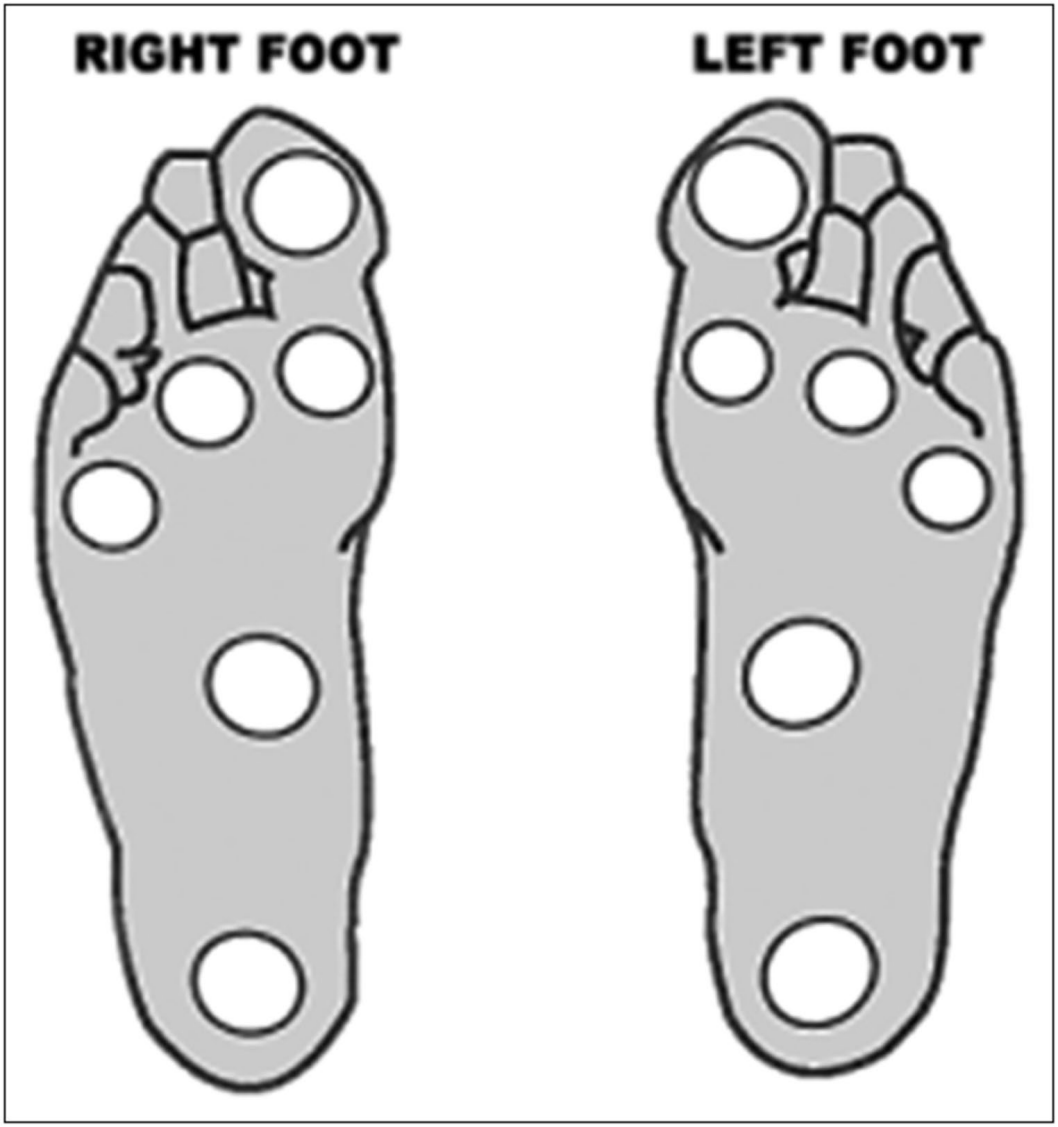
The sites of application of the biothesiometer probe [18]

As already mentioned, VPT was used as a screening tool for detecting early cases of DPN. VPT was measured using a biothesiometer. The probe was first applied to the patient’s hand to demonstrate the sense of vibration. The probe was then applied firmly and perpendicularly against six points on each foot namely: the big toe, base of the first, third, and fifth metatarsals, instep, and heel (Fig. 1). The voltage was escalated from 0 to 50 V until the patient feels the impulse. The process was repeated three times at each site on both feet. When the coefficient of variation (CV) exceeded 15%, extra readings were taken. The average of these readings was taken as the mean value for VPT.

Sensory nerve conduction properties, including sensory nerve conduction (SCV) velocity and action potential amplitude (SNAP), were elicited bilaterally in the sural nerve.

Motor nerve conduction velocity (MCV) and compound muscle action potential amplitude (CMAP) were measured bilaterally in the peroneal nerve. Surface electrodes and digital equipment were used for stimulation and recording. The test was done under strict standardized procedure, and the (CV) allowed did not exceed 3%; otherwise, additional readings were taken. Whenever necessary, the legs were warmed for ten minutes to ensure a skin temperature > 34°c before applying the test.

The average values measured for both (VPT) and conduction velocities were repeated in duplicates on two separate days (not more than a week apart) in the initial visit for all groups as baseline values, and three and six months later in the study groups (A) & (B). Sensory and motor nerve conduction studies were conducted using a Cadwell Sierra Wave 8155 system (Cadwell Sierra Wave 8155 certified to CAN/TSA EP/EMG measuring system-4 spaces channels version 08.11, USA), while VPT was measured with a Vibrameter-VPT biothesiometer (Somedic AB, Sweden).

### Sample size and statistical analysis

Based on a finding of the previous study [19], a difference of 5% in nerve conduction velocity (NCV) with a standard deviation of 3.9 m/s was considered a clinically meaningful difference between the alpha-amylase and the placebo groups. Using two-sided t-test, we estimated a sample size of 40 subjects per group, considering a power of 80% and alpha error rate of 5%.

Continuous variables were described as mean ± SD, while categorical variables were presented as counts and percentages. We used the Kruskal-Wallis test or one-way ANOVA based on a normality check to compare baseline characteristics. Normality testing of continuous variables was adopted using the Shapiro-Wilk test. Categorical variables were compared across the groups using the Pearson Chi-square test.

To evaluate longitudinal changes in neurophysiologic and biochemical outcomes, we used generalized estimating equation (GEE) models with an exchangeable correlation structure to account for within-subject repeated measures at baseline, 3 months, and 6 months. Robust sandwich variance estimators were used to obtain valid standard errors. Estimated marginal means and pairwise contrasts between treatment groups at each time point were calculated with Tukey adjustment for multiple comparisons.

A two-sided p-value of less than 0.05 was considered statistically significant. Missing data was omitted; therefore, no imputations were performed. All analyses were performed using R software (version 4.3.1, R Foundation for Statistical Computing).

## Results

**Fig. 2:**
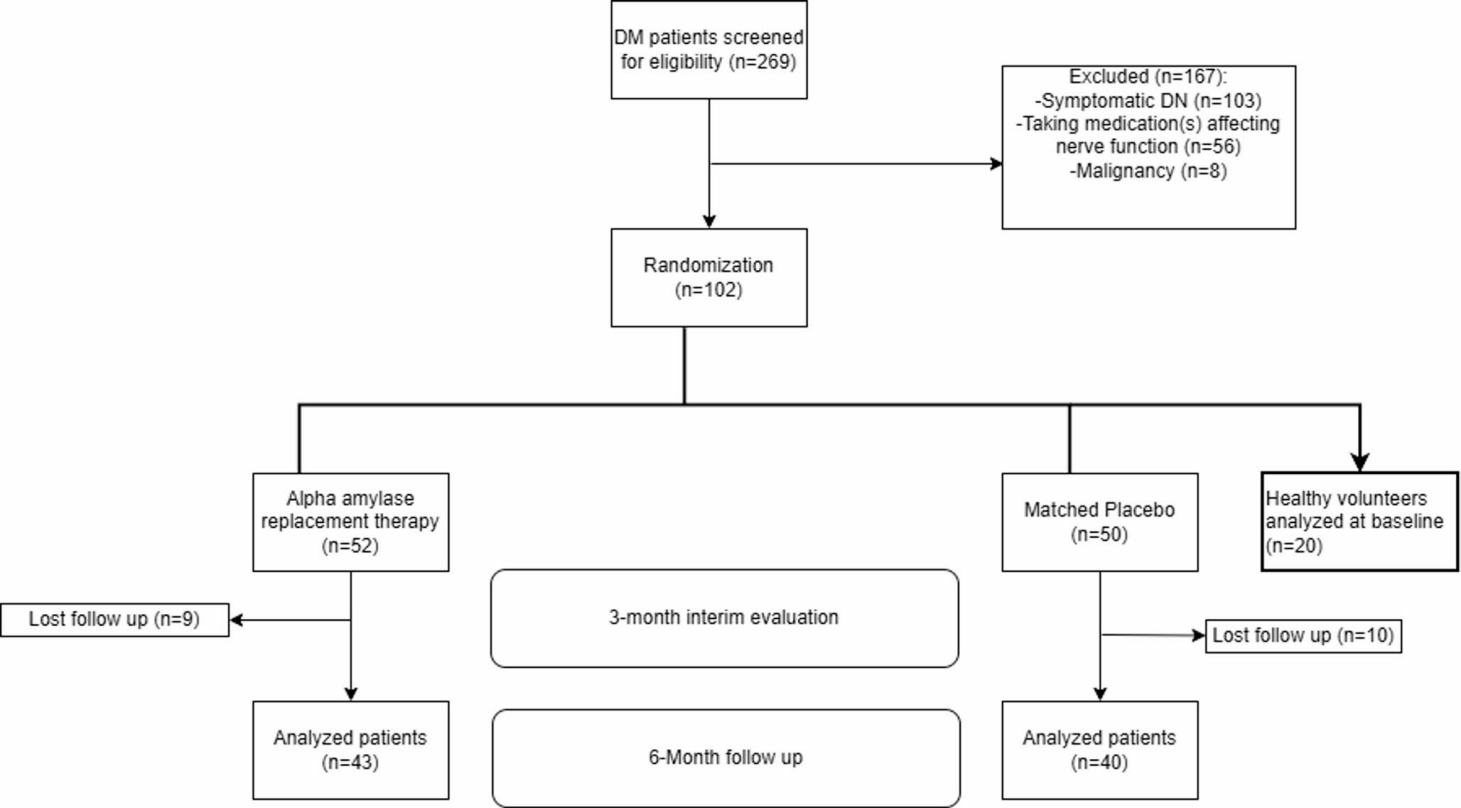
Study flow chart. Patients were followed for 6 months, with intermediate assessments at 3 months

A total of 83 patients were randomly allocated into two groups; the FDGL Gp A (43 patients) and the placebo Gp B (40 patients). The study flowchart is depicted in Fig. 2.

The characteristics of the 83 diabetic patients enrolled in the study are presented in Table 1. There were no statistically significant differences between the FDGL (α-amylase) group (Gp A; *n* = 43) and the placebo group (Gp B; *n* = 40) in any of the baseline demographic or clinical parameters. The mean age was 44.63 ± 7.07 years in Gp A and 45.35 ± 8.29 years in Gp B, while the mean BMI values were 28.09 ± 6.04 kg/m² and 26.97 ± 5.25 kg/m², respectively. The duration of diabetes was comparable between groups, averaging 4.75 ± 0.91 years in Gp A and 4.97 ± 1.08 years in Gp B (*p* = 0.27). Both groups included a comparable proportion of type 1 diabetic patients (14% vs. 15% in Gp A and Gp B, respectively; *p* > 0.9).

At baseline, the mean HbA₁C was 7.32 ± 0.59% in Gp A and 7.47 ± 0.39% in Gp B, with no significant difference between the two groups; however, both values were significantly higher than that of the control group (5.26 ± 0.53%, *p* < 0.001). Similarly, serum α-amylase activity was significantly reduced in both diabetic groups (39.09 ± 2.27 IU/L in Gp A and 38.67 ± 1.81 IU/L in Gp B) compared with the healthy control group (67.69 ± 1.95 IU/L, *p* < 0.001). No significant differences were observed between Gp A and Gp B in any of these parameters at baseline.

No adverse drug reactions or events related to the drug under trial were recorded during the study period, nor were there any significant changes in blood chemistry or vital signs or in blood chemistry except for 3 patients in group A. These patients (known hypertensives) noticed a persistent drop in their blood pressure after 2 months of treatment, which dictated a dose reduction of the hypotensive agent used (or exchange of the agent) to achieve a convenient pressure.

As regards NCV, the mean baseline values (Table 2) in the sural nerve (SCV) in the diabetic patients were 50 ± 2.5 m/s vs. 49.8 ± 2.7 m/s in the groups A & B, respectively, with no significant difference (*P* = 0.82). Yet their mean value differed significantly from the control value 53.9 ± 1.6 m/s (*p* < 0.001 for both groups).

After 3 and 6 months of treatment, there was a statistically significant increase in SCV (Table 3) in the amylase Gp A 2.73 ± 2.47 m/s compared with 0.16 ± 0.40 m/s in the placebo Gp B (*p* < 0.001). This difference increased further after 6 months in Gp A being 3.87 ± 2.36 m/s (amounting to 99% of the deficit from the control group; 53.9 ± 1.6 m/s) while it lingered at 0.54 ± 0.68 m/s in Gp B (*p* < 0.001).

As for motor conduction velocity (MCV) (Table 2), the mean values at baseline were 47.8 ± 3 and 48.8 ± 2.5 m/s in groups A & B, respectively, with no statistically significant difference (*p* = 0.06). Yet their mean value 48.3 ± 2.78 was significantly lower than the control group 52.5 ± 1.7 m/s (*p* < 0.001). After 3 months of treatment (Table 3) there was a statistically significant increase in the MCV in Gp A 1.15 ± 1.08 m/s compared with 0.28 ± 0.43 m/s (*p* < 0.01) in Gp B. This increase jumped even further after 6 months of treatment being 1.49 ± 1.18 m/s in Gp A compared with 0.17 ± 0.52 m/s in Gp B (*p* < 0.001). This increase in the Gp A amounted to 31.7% restoration of the deficit from the control.

As for SNAP (Table 2) the recorded baseline values were 4.99 ± 1.63, 4.77 ± 1.70 and 5.70 ± 1.09 µV for Gp A, Gp B, and control, respectively, with no significant difference among the groups at baseline (*p* = 0.2).

This goes also for CMAP where the values recorded were 3.99 ± 1.65, 3.85 ± 1.68, 4.85 ± 1.32 mV in Gp A, Gp B, and control, again with no statistically significant difference (*p* = 0.06, Table 2). Despite the significant increase in CMAP in the amylase group at 3 months (*p* = 0.025), the values recorded at the end of the study for the study groups were also statistically insignificant [*p* = 0.46] (Table 3).

As for VPT, (Table 2), the mean value recorded in the control group was 12.3 ± 2.1 V compared with 21 ± 4.6 and 19.7 ± 2.8 V in the Gp A and Gp B, respectively (*p* < 0.001). After 3 & 6 months in the study groups, the values were (14.07 ± 4.47 V and 13.73 ± 4.59 V) vs. (21.48 ± 2.86 V and 20.46 ± 3.38 V), respectively. This shows a final drop in Gp A VPT amounting to 7.27 V (83.6% of the deficit from the control group; *p* < 0.001) while the placebo group lingered around the same threshold. HbA1c demonstrated a modest reduction at 6 months in the FDGL group compared with placebo (− 0.19 ± 0.15% vs. − 0.10 ± 0.20%; *p* = 0.027), although no significant between-group difference was observed at 3 months.

The generalized estimating-equation (GEE) models suggested that alpha amylase group was associated with a significantly greater and consistent improvement over the 6 months in SCV, serum α-amylase concentrations, and VPT than placebo (Table S1). Significant group-by-time interaction terms were observed for MCV (β = 0.22 m/s per month; *p* < 0.001), SCV (β = 0.56 m/s per month; *p* < 0.001), serum amylase (β = 3.47 IU/L per month; *p* < 0.001), and VPT (β = −1.34 V per month; *p* < 0.001).

Pairwise least square mean differences further supported these findings (Table S2). At 6 months, FDGL therapy was associated with a mean improvement (vs. placebo) of + 3.91 m/s in SCV (*p* < 0.001), + 20.4 IU/L in serum amylase (*p* < 0.001), and a − 8.35 V reduction in VPT (*p* < 0.001). MCV improved modestly over time in both groups; however, between-group differences were not statistically significant at 3 or 6 months. A sensitivity analysis comparing the biochemical and electrophysiological changes between diabetes types 1 and 2 demonstrated statistically non-significant effects of the diabetes types on MCV, SCV, CMAP, VPT, SNAP, and serum amylase levels at both 3- and 6-month assessments. The reduction in HbA1C was significantly associated with type 1 DM at 3 months (*p* = 0.02); however, this effect did not persist to the 6-month assessment (*p* = 0.53).

**Table 1:** Baseline characteristics of the patient population.

Variable	Overall, *n* = 103^1^	α-Amylase (*n* = 43)^1^	Placebo (*n* = 40)^1^	Control (*n* = 20)^1^	*p* ^2^
**Age (yrs)**	45.58 ± 7.12	44.63 ± 7.07	45.35 ± 8.29	48.10 ± 3.43	0.19^2^
**Male gender (n**,** %)**	59 (57%)	24 (56%)	23 (58%)	12 (60%)	> 0.9^3^
**BMI (kg/m²)**	27.51 ± 5.36	28.09 ± 6.04	26.97 ± 5.25	27.34 ± 3.99	0.67^2^
**Diabetes type I (n**,** %)**	12 (14%)	6 (14%)	6 (15%)	NA	> 0.9^3^
**Diabetes duration (yrs)**	4.86 ± 1.00	4.75 ± 0.91	4.97 ± 1.08	NA ± NA	0.27^2^
**HbA1C (%)**	6.98 ± 0.99	7.32 ± 0.59^a^	7.47 ± 0.39^a^	5.26 ± 0.53^b^	**< 0.001** ^**2**^
**Serum α-amylase (IU/L)**	44.48 ± 11.63	39.09 ± 2.27^a^	38.67 ± 1.81^a^	67.69 ± 1.95^b^	**< 0.001** ^**2**^

^1^Data are means ± SD

^2^One-way ANOVA test

^3^Pearson’s Chi-squared test^4^Independent t-test.Values within a row sharing the same superscript letter (a, b) are not significantly different; values with different letters differ significantly (*p* < 0.05), based on Tukey’s post hoc test.BMI: body mass index; FDGL: fermented deglycyrrhizinated licorice; HbA1c: Glycated hemoglobin.

**Table 2:** Baseline values of neurophysiological and senso-motor variables.

Variable	Overall, *n* = 103^1^	α-Amylase (*n* = 43)^1^	Placebo (*n* = 40)^1^	Control (*n* = 20)^1^	*p* ^2^
**MCV (m/s)**	49.1 ± 3.1	47.8 ± 3.0^a^	48.8 ± 2.5^a^	52.5 ± 1.7^b^	**< 0.001**
**CMAP (mV)**	4.10 ± 1.63	3.99 ± 1.65	3.85 ± 1.68	4.85 ± 1.32	0.06
**SCV (m/s)**	50.7 ± 2.9	50.0 ± 2.5^a^	49.8 ± 2.7^a^	53.9 ± 1.6^b^	**< 0.001**
**SNAP (µV)**	5.04 ± 1.59	4.99 ± 1.63	4.77 ± 1.70	5.70 ± 1.09	0.20
**VPT (V)**	18.8 ± 4.8	21.0 ± 4.6^a^	19.7 ± 2.8^a^	12.3 ± 2.1^b^	**< 0.001**

^1^Data are means ± SD

^2^One-way ANOVAValues within a row sharing the same superscript letter (a, b) are not significantly different; values with different letters differ significantly (*p* < 0.05), based on Tukey’s post hoc test.CMAP: compound muscle action potential; FDGL: fermented deglycyrrhizinated licorice; MCV: motor nerve conduction velocity; SCV: sensory nerve conduction velocity; SNAP: sensory nerve action potential; VPT: vibration perception threshold.

**Table 3:** Longitudinal changes in neurophysiological and senso-motor parameters in the alpha amylase group compared to placebo.

Variable	3 Months			6 Months		
	α-Amylase (*n* = 43)^1^	Placebo (*n* = 40)^1^	*p* ^2^	α-Amylase (*n* = 43)^1^	Placebo (*n* = 40)^1^	*p* ^2^
**ΔMCV (m/s)**	1.15 ± 1.08	0.28 ± 0.43	**< 0.001**	1.49 ± 1.18	0.17 ± 0.52	**< 0.001**
**ΔCMAP (mV)**	0.19 ± 0.88	-0.16 ± 0.63	**0.025**	0.60 ± 0.70	0.52 ± 0.25	0.458
**ΔSCV (m/s)**	2.73 ± 2.47	0.16 ± 0.40	**< 0.001**	3.87 ± 2.36	0.54 ± 0.68	**< 0.001**
**ΔSNAP (µV)**	0.39 ± 0.35	0.12 ± 1.20	0.175	0.46 ± 0.40	0.43 ± 0.32	0.756
**ΔVPT (V)**	-6.93 ± 1.94	1.78 ± 0.78	**< 0.001**	-7.27 ± 1.35	0.76 ± 2.11	**< 0.001**
**ΔHbA1C**	-0.12 ± 0.29	-0.18 ± 0.22	0.32	-0.19 ± 0.15	-0.10 ± 0.20	**0.027**
**ΔSerum amylase**	8.73 ± 2.38	0.77 ± 0.15	**< 0.001**	20.74 ± 3.38	-0.11 ± 0.13	**< 0.001**

^1^Mean ± SD

^2^Independent t-test testCMAP: compound muscle action potential; FDGL: fermented deglycyrrhizinated licorice; HbA1c: glycated hemoglobin; MCV: motor nerve conduction velocity; SCV: sensory nerve conduction velocity; SNAP: sensory nerve action potential; VPT: vibration perception threshold

**Table 4:** Comparing changes in neurophysiological and sensorimotor parameters across diabetes types.

	**Type 1 Diabetes**			**Type 2 Diabetes**			
**Characteristic**	**FDGL Group** *n* = 6^1^	**Placebo Group** *n* = 6^1^	**p-value** ^2^	**FDGL Group** *n* = 37^1^	**Placebo Group** *n* = 34^1^	**p-value** ^2^	**p-interaction** ^3^
3-month							
ΔMCV (m/s)	0.90 ± 0.92	0.32 ± 0.27	0.2	1.19 ± 1.11	0.28 ± 0.45	**< 0.001**	0.53
ΔCMAP (mV)	2.48 ± 2.28	0.07 ± 0.52	**0.048**	2.77 ± 2.53	0.17 ± 0.38	**< 0.001**	0.85
ΔSCV (m/s)	-7.63 ± 1.65	1.85 ± 0.93	**< 0.001**	-6.82 ± 1.98	1.77 ± 0.77	**< 0.001**	0.87
ΔSNAP (µV)	0.33 ± 0.17	0.45 ± 1.04	0.8	0.39 ± 0.37	0.07 ± 1.23	0.14	0.41
ΔVPT (V)	-0.03 ± 1.11	-0.33 ± 0.59	0.6	0.26 ± 0.85	-0.13 ± 0.64	**0.029**	0.35
ΔHbA1C	0.02 ± 0.33	-0.37 ± 0.12	**0.036**	-0.15 ± 0.28	-0.15 ± 0.22	> 0.9	**0.02**
ΔSerum amylase	7.33 ± 1.04	0.78 ± 0.19	**< 0.001**	8.95 ± 2.46	0.76 ± 0.15	**< 0.001**	0.12
6-month							
ΔMCV (m/s)	1.03 ± 1.16	0.35 ± 0.81	0.3	1.56 ± 1.18	0.14 ± 0.47	**< 0.001**	0.20
ΔCMAP (mV)	4.42 ± 1.65	1.07 ± 0.67	**0.003**	3.78 ± 2.47	0.45 ± 0.64	**< 0.001**	0.08
ΔSCV (m/s)	-7.07 ± 1.39	-0.87 ± 2.02	**< 0.001**	-7.30 ± 1.36	1.05 ± 2.02	**< 0.001**	0.99
ΔSNAP (µV)	0.55 ± 0.35	0.36 ± 0.10	0.3	0.44 ± 0.41	0.45 ± 0.34	> 0.9	0.42
ΔVPT (V)	1.09 ± 0.59	0.51 ± 0.33	0.069	0.52 ± 0.69	0.52 ± 0.24	> 0.9	0.05
ΔHbA1C	-0.25 ± 0.10	-0.10 ± 0.15	0.082	-0.18 ± 0.16	-0.10 ± 0.21	0.083	0.53
ΔSerum amylase	20.05 ± 3.64	-0.18 ± 0.16	**< 0.001**	20.85 ± 3.37	-0.10 ± 0.12	**< 0.001**	0.64

^1^Mean ± SD

^2^Welch Two Sample t-test^3^two-way ANOVACMAP: compound muscle action potential; FDGL: fermented deglycyrrhizinated licorice; HbA1c: glycated hemoglobin; MCV: motor nerve conduction velocity; SCV: sensory nerve conduction velocity; SNAP: sensory nerve action potential; VPT: vibration perception threshold

## Discussion

α-amylase enzyme deficiency has been recently demonstrated in diabetic patients and correlates with complication severity [6–8, 17]. Thus, treating diabetic patients with this enzyme would be a kind of replacement therapy. Therefore, the timing of intervention is expected to have a great impact on the outcome [20] i.e., the earlier the intervention, the better the results. For this reason, the patients of this study were carefully selected with an average diabetic duration of less than 5 years without clinical DPN symptoms; yet, having demonstrable significant sensory and motor conduction deficits (when compared with controls of relevant size and age) (Table 1). This is because, at this phase, the early sensory-motor nerve conduction deficits are likely to reflect early circulatory and metabolic disturbances rather than structural changes that could have multifactorial causes [20, 21] and possibly not amenable to reversibility.

In the present study, NCV improved significantly in both sensory and motor nerves in Gp A, rising steadily during the study period. After 6 months, SCV improvement amounted to about 99% of the deficit for the control (*p* < 0.001). VPT assessment, which was also carried along the sural nerve, was significantly reduced within 6 months in Gp A by about 83.6% of the deficit from control (*P* < 0.001). This confirms the full restoration of the sensory nerve functions with the use of the drug formulation. As already mentioned, the pathogenesis of DPN involves early vascular and metabolic changes, which result in nerve ischemia along with osmotic stress due to excessive formation of sorbitol secondary to intracellular hyperglycemia [11]. Reversing these early changes can be accomplished with antioxidants [22]. However, the antioxidant effect of flavonoids alone can hardly account for that, since a similar effect was not noticed in Gp B, which received DGL.

On the other hand, MCV, which reflects true structural neuronal damage due to the formation of intraneural advanced glycation end products and ROS, increased significantly in GpA compared to GpB (*P* < 0.001). This increase amounted to about 32% of the deficit from the control. Again, neither the aldose reductase inhibition nor the antioxidant effect of flavonoids present in licorice [22] could account for the neural regeneration reflected by improved MCV in Gp A, since patients in Gp B did not experience such an effect. The only remaining factor that can account for the changes in Gp A is the presence of α-amylase enzyme in FDGL, whose probable mechanism of action involves proper utilization of carbohydrates through the promotion of glycolysis, and hence, reducing intracellular glucose concentration.

As for SNAP and CNAP, they showed no baseline differences between groups and controls, and they were hence disregarded. The statistically insignificant difference of HBA1C between the study groups, whether at baseline (Table 1) or after 6 months (Table 3), strongly suggests that it could not account for the improved neural function at the end of the study.

The steady rise of serum amylase in Gp A after 3 and 6 months (*P* < 0.001 for both) compared with a drop in Gp B proves the ability of the drug to cause a cumulative rise in serum amylase over time. This is quite an achievement because the absorption of enzymes is guarded by the immune cells embedded among the intestinal cells of the mucosa to safeguard against the absorption of antigenic proteins that may cause anaphylaxis. Up to 90% of the given dose of an oral enzyme might be lost in this way [3].

Interestingly, we investigated whether the therapeutic response to FDGL differed between patients with type 1 and type 2 diabetes. Although type 1 diabetes constitutes a smaller proportion of the cohort (14%), subgroup analyses demonstrated consistent improvements in MCV and SCV, VPT, and serum amylase levels in both diabetes types following FDGL therapy. No differences between both diabetes types were observed for any neurophysiological parameter at either 3 or 6 months. A transient reduction of HbA1c at 3 months was noted in type 1 DM, but did not persist at 6 months, further supporting that the observed neurological benefit is unlikely to be driven by glycemic change. This could be attributed to the better absorption of amylase in the non-aged gastrointestinal tract of the younger patients with DM1.

Pancreatic amylase is secreted into the gut lumen and reabsorbed by intestinal cells [23] through macromolecular protein absorption. Previous studies proposed an entero-pancreatic recycling of the enzyme [4, 5]. However, other studies failed to retrieve an appreciable amount of the enzyme in the pancreas, with a high concentration of the enzyme (18% – 25%) in the liver [24, 25].

On the other hand, glycogen phosphorylase (GPH) is another glycolytic enzyme present in the liver and all body cells for glycogenolysis. Since both enzymes act on a chain of glucose molecules bound by what is known as 1–4 glycosidic bonds, their catalytic and binding sites must be identical. Structural comparison of both enzymes demonstrated that GPH (97.43 KDa, 842 amino acids) [26, 27] is approximately twice in size of α-amylase (55.8 KDa, 496 amino acids). GPH is a dimer made up of two identical subunits with a catalytic and binding site on each subunit. This suggests (according to the law of conservation of energy) that the liver incorporates two molecules of amylase to form one molecule of GPH. Two additional sites are added by the liver to the pancreatic amylase: an allosteric site and a phosphorylating site. Since they are also found in duplicates, it entails that they had been added before the final assembly of the GPH molecule. Perhaps this is the enzyme known as hepatic amylase, which has no glycolytic activity in the liver, but is just a transitional stage [27, 28] that is sent to the distant tissues to form GPH. This explains that the liver is the major source of serum amylase [29] and that there is no trace of GPH in the serum.

Pushing this theory a bit further, let us now focus on two more enzymes: glucokinase and its isomer, hexokinase. Both enzymes provide the hexose molecule with a phosphate group to start glycolysis (the preliminary step for the breakdown of hexoses in the cytosol). This step is known to be the rate-limiting step for glycolysis. Glucokinase is present in the liver and pancreas, whereas hexokinase is present in all other body cells. If the liver is capable of mounting a phosphorylating site on the amylase molecule (as already suggested), then glucokinase (and hence its isomer hexokinase) is but a derivative of amylase with a molecular wt. 52 kDa & AA residues 465 [30], after expelling the active site.

Putting the pieces together, it is suggested that α-amylase is the precursor of the three phosphorylating enzymes; (GPH), glucokinase, and hexokinase, which are the bottleneck of glycolysis since phosphorylation of the glucose molecule is the rate-limiting step of glycolysis. Not only is glycolysis the preliminary and mandatory step for the further breakdown of carbohydrates in the mitochondria to yield energy, but it is also the sole source of energy for red blood cells and a major source of energy for white blood cells, the cornea, lens, retina, testes, ova, renal medulla, and heavily exercising muscles. Therefore, the lack of energy production in these sites contributes to the complications occurring in diabetes mellitus.

It goes without saying that promoting glycolysis will abort such complications. As for DPN, it has been mentioned already that intracellular hyperglycemia is the cause of neural damage. Promoting glycolysis by α-amylase replacement will not only reduce intracellular hyperglycemia but will also provide the mitochondria with ample amounts of pyruvate (the end product of glycolysis). This pyruvate is translocated to mitochondria to produce oxaloacetate and acetyl-CoA, which unite to form citrate, thereby igniting the TCA cycle to provide enough energy for cellular repairs. This energy is augmented by the aldose reductase inhibition [31] and the antioxidant effect of flavonoids found in licorice [22].

By now, it is possible to answer the original question of what links amylase with the pathogenesis of DM. The pathogenic process resulting from the impaired carbohydrate metabolism in diabetic patients starts with hypoamylasemia in the prediabetic phase. This puts a huge burden on the islet cells, which struggle to achieve normoglycemia in the face of an ever-rising hypoamylasemia by producing excessive amounts of insulin in the prediabetic phase. The normal regeneration rate of the islet cells (around 3%) will not be enough to compensate for the excessive loss. When the islet cell mass eventually drops to around 40%-50%, frank hyperglycemia will ensue [32]. In other words, hyperglycemia does not represent the onset of DM, but it heralds the failure of the combined effort of the liver and pancreas to maintain normoglycemia. This explains why diabetic complications like hypertension, dyslipidemia, or coagulopathy may appear much earlier than the onset of hyperglycemia in the insulin resistance syndrome.

Thus, it can be deduced from the above that supplementation of α amylase enzyme to diabetic patients is expected to promote glycolysis, hence restoring the normal metabolic pathway of carbohydrates and accordingly interrupting the polyol pathway (which produces sorbitol responsible for many diabetic complications) as well as furnishing the tissues with an ample amount of energy from carbohydrate breakdown. This promotion of cellular energy production can explain why intravenous infusion of amylase reduces the overproduction of insulin and C-peptide, without influencing glucose tolerance in response to an IV glucose challenge [33].

To our knowledge, no previous human clinical trial has evaluated FDGL in diabetic neuropathy. However, prior animal studies demonstrated a significant histological recovery of corneal, neural, muscular, and renal tissues in diabetic rats treated with the same FDGL formulation [14–16]. Oral amylase treatment of diabetic rats caused amelioration and regression of the diabetic insult of the cornea (one of the tissues almost totally dependent on glycolysis for its energy production [15]. Also, histocytological examination of the gastrocnemius and sciatic nerve of diabetic rats revealed restoration of manual structure with oral amylase replacement [14]. In another study, the drug formulation containing amylase succeeded in ameliorating the adverse effects of DM on the structure of the renal cortex in diabetic rats [16]. The significant improvement of histological parameters in animal models with retinopathy and nephropathy suggests that these microvascular complications share common pathogenic mechanisms with neuropathy, which appear to be mitigated by FDGL treatment.

The current study findings should be interpreted cautiously. To avoid potential confounding with our study outcomes, insulin regimens and oral hypoglycemics were kept stable throughout the 6-month period, and any dose adjustments were documented. Sensitivity analyses indicated that FDGL effects were consistent across both type 1 and type 2 DM. However, important variables such as diet and physical activity could not be fully controlled and may have varied among participants, representing potential residual confounders. Furthermore, the single-center nature of the current study may limit the generalizability of our findings. Future studies should be conducted in multiple centers, with strict study protocols to control for the participants’ dietary intake and level of physical activity.

To sum up, whether the proposed relationship between amylase and phosphorylating enzymes represents fact or hypothesis, hypoamylasemia remains consistent in diabetic patients and correlates with complication severity. However, our study is limited by short-term follow-up; therefore, the current findings need to be validated in longer-term studies.

## Conclusion

From this study, it can be concluded that FDGL comprising α - amylase, acid lipase, and flavonoids naturally present in licorice root extract, can promote the reversal of early DPN. Further studies are certainly required to confirm the ability of this drug to reverse or at least abort the progression of diabetic complications, especially when given early in the course of the disease.

## Supplementary Information

Below is the link to the electronic supplementary material.

Supplementary Material 1

## Data Availability

The dataset is available from the corresponding author on reasonable request.

## Abbreviations

AA: Amino Acids
BMI: Body Mass Index
CMAP: Compound Muscle Action Potential
CV: Coefficient of Variation
DGL: Deglycyrrhizinated Licorice
DM: Diabetes Mellitus
DPN: Diabetic Polyneuropathy
ECG: Electrocardiogram
FDGL: Fermented Deglycyrrhizinated Licorice
GPH: Glycogen Phosphorylase
HbA1c: Glycated Hemoglobin
IU: International Units
MCV: Motor Nerve Conduction Velocity
NCV: Nerve Conduction Velocity
RBC: Red Blood Cells
SCV: Sensory Nerve Conduction Velocity
SD: Standard Deviation
SNAP: Sensory Nerve Action Potential
TCA: Tricarboxylic Acid
VPT: Vibration Perception Threshold
WBC: White Blood Cells
