# Supplementary material for: Randomized Double-blind Placebo-controlled study to evaluate the efficacy of fermented deglycyrrhizinated licorice for treatment of diabetic polyneuropathy
Source: Endocrine. 2026 Feb 2;91(1):51. doi: 10.1007/s12020-025-04476-5 (PMC12864363; doi:10.1007/s12020-025-04476-5)
Supplement: Supplementary file 1 — Supplementary Material 1 [file 12020_2025_4476_MOESM1_ESM.docx]

**Table (S1): Generalized estimating equation models for longitudinal changes in neurophysiologic and biochemical Outcomes.**

**(A) MCV model**

| **Predictor** | **Estimate** | **Std.Err** | **Wald** | **p** |
| --- | --- | --- | --- | --- |
| Intercept (Placebo at baseline) | 48.984 | 0.339 | 20925.04 | <0.001* |
| α-amylase (vs Placebo) | −1.063 | 0.510 | 4.34 | 0.037* |
| Time (months) | 0.028 | 0.014 | 4.21 | 0.040* |
| α-amylase × Time | 0.220 | 0.033 | 45.44 | <0.001* |

**(B) SCV model**

| **Predictor** | **Estimate** | **Std.Err** | **Wald** | **p** |
| --- | --- | --- | --- | --- |
| Intercept (Placebo at baseline) | 49.712 | 0.425 | 13683.0 | <0.001* |
| α-amylase (vs Placebo) | 0.583 | 0.583 | 1.00 | 0.320 |
| Time (months) | 0.090 | 0.018 | 26.20 | <0.001* |
| α-amylase × Time | 0.555 | 0.062 | 80.50 | <0.001* |

**(C) Serum amylase model**

| **Predictor** | **Estimate** | **Std.Err** | **Wald** | **p** |
| --- | --- | --- | --- | --- |
| Intercept (Placebo at baseline) | 38.939 | 0.285 | 18709.25 | <0.001* |
| α-amylase (vs Placebo) | −0.401 | 0.456 | 0.78 | 0.380 |
| Time (months) | −0.018 | 0.003 | 31.43 | <0.001* |
| α-amylase × Time | 3.475 | 0.085 | 1673.55 | <0.001* |

**(D) VPT model**

| **Predictor** | **Estimate** | **Std.Err** | **Wald** | **p** |
| --- | --- | --- | --- | --- |
| Intercept (Placebo at baseline) | 20.180 | 0.441 | 2096.88 | <0.001* |
| α-amylase (vs Placebo) | −0.322 | 0.804 | 0.16 | 0.689 |
| Time (months) | 0.127 | 0.055 | 5.33 | 0.021* |
| α-amylase × Time | −1.338 | 0.065 | 429.77 | <0.001* |

MCV: motor nerve conduction velocity; SCV: sensory nerve conduction velocity; VPT: vibration perception threshold.

**Table (S2): Least-square (LS) means differences between FDGL and placebo over time.**

**(A) MCV**

| **Time (months)** | **Difference (LS means)** | **SE** | **z** | **95% CI** | **p^1^** |
| --- | --- | --- | --- | --- | --- |
| 0 | −1.063 | 0.510 | −2.083 | [−2.063, −0.063] | 0.037* |
| 3 | −0.402 | 0.513 | −0.784 | [−1.407, 0.603] | 0.433 |
| 6 | 0.258 | 0.534 | 0.483 | [−0.789, 1.305] | 0.629 |

**(B) SCV**

| **Time (months)** | **Difference (LS means)** | **SE** | **z** | **95% CI** | **p^1^** |
| --- | --- | --- | --- | --- | --- |
| 0 | 0.580 | 0.583 | 1.000 | [−0.563, 1.723] | 0.318 |
| 3 | 2.250 | 0.577 | 3.900 | [1.119, 3.381] | <0.001* |
| 6 | 3.910 | 0.628 | 6.230 | [2.679, 5.141] | <0.001* |

**(C) Serum amylase**

| **Time (months)** | **Difference (LS means)** | **SE** | **z** | **95% CI** | **p^1^** |
| --- | --- | --- | --- | --- | --- |
| 0 | −0.400 | 0.456 | −0.900 | [−1.294, 0.494] | 0.379 |
| 3 | 10.000 | 0.463 | 21.700 | [9.093, 10.907] | <0.001* |
| 6 | 20.400 | 0.592 | 34.500 | [19.240, 21.560] | <0.001* |

**(D) VPT**

| **Time (months)** | **Difference (LS means)** | **SE** | **z** | **95% CI** | **p^1^** |
| --- | --- | --- | --- | --- | --- |
| 0 | −0.320 | 0.804 | −0.400 | [−1.896, 1.256] | 0.689 |
| 3 | −4.340 | 0.797 | −5.440 | [−5.902, −2.778] | <0.001* |
| 6 | −8.350 | 0.836 | −9.990 | [−9.989, −6.711] | <0.001* |

^1^P values adjusted using Tukey’s post hoc test

CI: confidence interval; FDGL: fermented deglycyrrhizinated licorice; MCV: motor nerve conduction velocity; SCV: sensory nerve conduction velocity; VPT: vibration perception threshold.
